# Efficacy of neuroendoscopic surgery versus craniotomy for supratentorial hypertensive intracerebral hemorrhage: A meta‐analysis of randomized controlled trials

**DOI:** 10.1002/brb3.1471

**Published:** 2019-11-19

**Authors:** Xu‐Hui Zhao, Su‐Zhen Zhang, Jin Feng, Zhen‐Zhong Li, Zeng‐Lu Ma

**Affiliations:** ^1^ Department of Neurosurgery Xingtai People's Hospital Xingtai China; ^2^ Clinical Laboratory Xingtai People's Hospital Xingtai China; ^3^ Department of Neurosurgery The Frist Hospital of Xingtai Xingtai China

**Keywords:** craniotomy, hypertensive intracerebral hemorrhage, meta‐analysis, neuroendoscopic surgery

## Abstract

**Background:**

Hypertensive cerebral hemorrhage (HCH) is a potentially life‐threatening neurological condition with an extremely high morbidity and mortality. In recent years, neuroendoscopy has been used to treat intracerebral hemorrhage (ICH). However, the choice of neuroendoscopic surgery versus craniotomy for patients with intracerebral hemorrhages is controversial.

**Aim:**

We conducted this meta‐analysis to assess the efficacy of neuroendoscopic surgery compared with craniotomy in patients with supratentorial hypertensive ICH.

**Methods:**

A systematic electronic search was conducted of online electronic databases: PubMed, Embase, and the Cochrane Library updated on December 2017. The meta‐analysis only included randomized controlled studies.

**Results:**

Three randomized controlled trials met our inclusion criteria. The pooled analysis of death showed that neuroendoscopic surgery decreased the rate of death when compared with craniotomy (RR = 0.58, 95% CI 0.26–1.29; *p* = .18). The pooled result of complications indicated that neuroendoscopic surgery has a tendency toward lower complications (RR = 0.37, 95% CI 0.28–0.49; *p* < .001).

**Conclusions:**

Our results suggested that neuroendoscopic surgery has lower complications, but no superior advantages in morbidity rates. Since the advantage of neuroendoscopic surgery has been performed in some area, the continuation of multi‐center comparative investigation with craniotomy may be necessary. Moreover, some efforts need to be taken in selecting appropriate patients with different treatments.

## Graphical abstract

Neuroendoscopic surgery significantly reduces the rate of complications in patients with HICH, when compared with craniotomy, while without improving death outcomes.

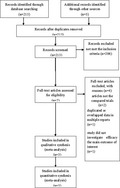

## 1 INTRODUCTION

Hypertensive intracerebral hemorrhage (HICH) is a condition that may threaten the life of patients with a high mortality rate (Hemphill et al., 2015) and severe disabilities (Asch et al., 2010; Mayer & Rincon, 2005) . The management of primary intracerebral hemorrhage (ICH) remains controversial. Previous studies have shown that surgical treatment rather than conservative treatment was associated with better outcomes (*p* < .001) (Mendelow et al., 2013) for HICH. For the treatment of ICH, surgical procedures have been employed. However, the effect was weak due to heterogeneity among the studies. The studies included different strategies of surgical interventions, such as craniotomy and neuroendoscopic surgery (Batjer, Reisch, Allen, Plaizier, & Su, 1990; Bhattathiri, Gregson, Prasad, Mendelow, & STICH Investigators, 2006; Teernstra et al., 2003; Xi et al., 1998).

In the past several decades, craniotomy has played critical roles for HICH. However, several prospective randomized controlled trials have failed to show benefit in outcome in craniotomy patients (Mendelow et al., 2005; Teernstra et al., 2003). Endoscopic surgery has been applied for the treatment of HICH in recent years. Many studies suggested that endoscopic evacuation showed efficacy and safety benefit for HICH patients (Cho, Chen, Chang, Lee, & Tso, 2006; Nagasaka et al., 2011), when compared with traditional craniotomy (Wang et al., 2015; Yamashiro, Hitoshi, Yoshida, & Kuratsu, 2015). The result is in accordance with the latest systemic review (Xia et al., 2018). However, due to retrospective research or limited sample size, no conclusion could be drawn about the effects of endoscopic surgery on outcomes in HICH patients (Auer et al., 1989; Xu et al., 2018). Therefore, due to these controversial results, we performed this meta‐analysis to identify which type of surgical procedure was safer and more effective in promoting outcomes and reducing complications in patients with HICH.

## 2 METHODS

### 2.1 Retrieval strategy

Published articles on the efficacy and safety of neuroendoscopic surgery compared with craniotomy in patients with supratentorial HICH up to December 2017 were retrieved. The searched databases included PubMed, Embase, and the Cochrane Library. The process was established to find all articles based on the MeSH terms and the keywords: “craniotomy”, “neuroendoscopic surgery” and “hypertensive intracerebral hemorrhage”, and no limitation was used during the literature search. We identified full‐text papers from reference materials for further evaluation.

### 2.2 Eligibility criteria

Articles that met the following inclusion criteria were included in this analysis: (a) studies were designed as randomized controlled trials; (b) studies enrolled HICH patients; (c) trials compared craniotomy versus neuroendoscopic surgery; and (d) studies provided data of perioperative morbidity or mortality. Studies that did not meet the above inclusion criteria were excluded from the meta‐analysis.

### 2.3 Quality assessment

Two investigators independently rated the quality of the retrieved studies. We chose the risk of bias items (ROBI) recommended by The Cochrane Handbook for Systematic Reviews of Interventions.

### 2.4 Data extraction

Data were extracted by two authors independently. Disagreement was resolved by consensus. From each of the eligible studies, the main categories were based on the following: first author's family name, publication year, total number of study subjects, mean age, hematoma volume (ml), and the number of mortalities.

### 2.5 Statistical analysis

Meta‐analysis was performed by pooling the results of the reported incidence of death and complications. Results were expressed as the appropriate ratio/difference for dichotomous outcomes as determined by available data. The *I* ^2^ statistic test was performed to further examine statistical heterogeneity between the trials (Higgins & Thompson, 2002). Studies with an *I* ^2^ ≥ 50% were considered to indicate moderate and high heterogeneity, and *I* ^2^ < 50% was considered to have low heterogeneity, respectively (Higgins, Thompson, Deeks, & Altman, 2003). The random‐effects model was adopted if *I* ^2^ > 50%; otherwise, the fixed‐effects model was chosen.

A *p* value < .05 was considered to be statistically significant. All the meta‐analyses were performed by Review Manager version 5.3 software (Revman; The Cochrane Collaboration, Oxford, United Kingdom). The findings of our meta‐analysis were shown in forest plots. The risk of bias was evaluated using Begg's test and Egger's test.

### 2.6 Ethical approval

Ethical approval was waived because this study did not involve any human participants or animals.

## 3 RESULTS

### 3.1 Overview of literature search and study characteristics

**Figure 1:**
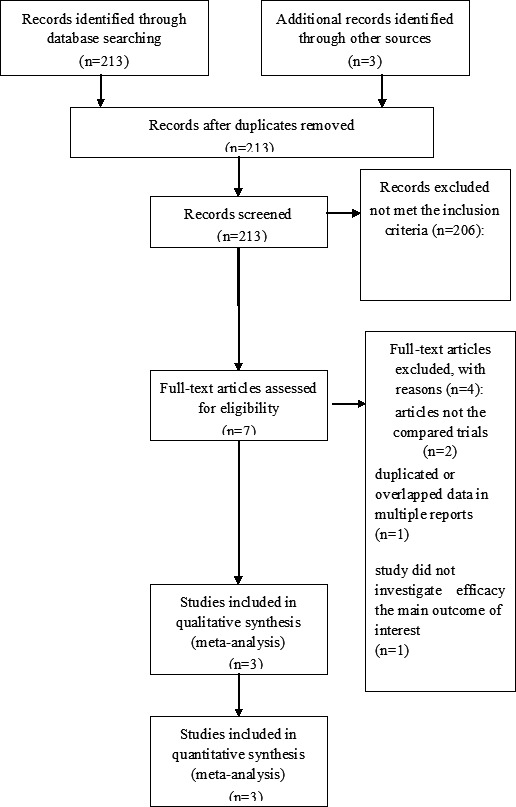
The PRISMA flowchart of the selection process to identify studies eligible for pooling

A total of 213 studies were initially identified after the primary selection. Based on the criteria described in the methods, 206 irrelevant citations were further excluded based on review of titles and abstracts. Finally, a total of 3 RCTs (Cho et al., 2006; Feng, He, Liu, Yang, & Wang, 2016; Zhang et al., 2014) were included in this meta‐analysis (Figure 1). The major characteristics of included studies are depicted in Table 1.

**Table 1:** The primary characteristics of the eligible studies in more detail

Author	Years	Total		M/F		Mean age		Hematoma volume (ml)		Death	
		NE	C	NE	C	NE	C	NE	C	NE	C
Feng Y	2016	93	91	56/37	58/33	66.35	69.1			8	8
Zhang HZ	2014	21	24	16/5	22/8	59.9	61.45	58.28	62.16	0	3
Cho DY	2006	30	30	19/11	21/9	56.67	54.22	55.48	42.11	0	4

### 3.2 Clinical and methodological heterogeneity

#### 3.2.1 Pooled analysis of death after neuroendoscopic surgery versus craniotomy

**Figure 2:**
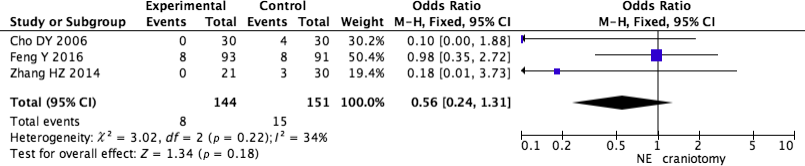
Pooled analysis of death after neuroendoscopic surgery versus craniotomy

The pooled analysis (Cho et al., 2006; Feng et al., 2016; Zhang et al., 2014) revealed no statistically difference in death between neuroendoscopic surgery and craniotomy (RR = 0.58, 95% CI 0.26–1.29; *p* = .18) (Figure 2).

#### 3.2.2 Pooled analysis of complications after neuroendoscopic surgery versus craniotomy

**Figure 3:**
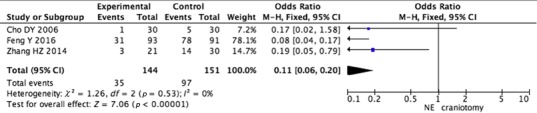
Pooled analysis of complications after neuroendoscopic surgery versus craniotomy

The random‐effects model was used to pool data on complications (Cho et al., 2006; Feng et al., 2016; Zhang et al., 2014). The pooled data showed that neuroendoscopic surgery had a lower risk of complications (RR = 0.37, 95% CI 0.28–0.49; *p* < .001) (Figure 3).

## 4 DISCUSSION

Hypertensive intracerebral hemorrhage (HICH) inflicts a major health burden with an extremely high morbidity and mortality. In case of a massive hematoma, surgical drainage is a crucial therapeutic option. Endoscopic evacuation has been investigated by comparison with traditional craniotomy (Cho et al., 2006). However, many questions regarding the minimally invasive surgery remain unanswered. Historically, craniotomy has been used as an appropriate therapy for HICH evacuation (Bosel, Zweckberger, & Hacke, 2015). A recent RCT showed that early craniotomy could reduce the mortality of ICH patients (Mendelow et al., 2013). Craniotomy had also some other advantages such as good view and immediate removal of hematoma and improvement in local blood circulation (Ohwaki et al., 2006), which could also help in outcomes. However, the therapies appear to have reached a plateau. Because of lack of large multi‐centric RCTs (Hemphill et al., 2015), many surgical trials have shown that craniotomy is associated with substantial adverse effects (Lee et al., 2003; Li, Yang, Xu, Li, & You, 2013; Prasad, Browman, Srivastava, & Menon, 1997). Craniotomy increased the operative time and the risk of infection. Therefore, minimally invasive surgery that causes minimal trauma to normal brain region during removal of hematoma has been under intensive investigation for treating ICH over craniotomy (Li et al., 2017).

In neuroendoscopic surgery, a small burr hole is created, a minimally invasive procedure in which a 5‐ to 8‐mm‐diameter endoscope is inserted into the brain tissue (Xu et al., 2018). The minimal invasive surgery is performed under a surgical microscope, which has the advantage of adequate hemostasis and small bone window craniotomy. Neuroendoscopic surgery, as the minimal invasive surgery, was successfully applied for hematoma evacuation with many advantages (Zhou et al., 2011). Some scholars have emphasized that site of hemorrhage, hemorrhage volume, and patient condition should be considered in the selection of surgical method (Li & Chen, 2014; Luo et al., 2008). Appropriate operative route is the key for success to treat SICH. The endoscopic removal of intracranial hematoma is proceeded within the cavity, which has made it possible to carry out minimally invasive interventions in brain tissues whether it is in the normal area or the surrounding damaged region (Zhang et al., 2014).

With regard to the incidence of complications, we found that patients that underwent neuroendoscopic surgery had fewer complications than those that underwent craniotomy. The neuroendoscopic surgery group had a reduced infection rate; the reasons for this benefit are multiple and include the following: (a) neuroendoscopic surgery provides multi‐angle observation and “observe around the corner” capability to manage intraoperative bleeding, which make up for the insufficiency of direct vision (Feng et al., 2016). (b) In some studies, to avoid brain tissue damage, some authors selected short and precise routes to the hematoma under direct vision and deep lesions without manipulating or exposing the unaffected areas (Zhang et al., 2014).

In conclusion, our results suggest that neuroendoscopic surgery significantly reduces the rate of complications in patients with HICH compared with craniotomy, while without improving death outcomes. When all these data were analyzed, it became obvious that these two methods had their own advantages and shortcomings, and every approach had its indications, so it was difficult to decide which one was better for HICH patients. Thus, it is an essential issue to select individualized treatment that will benefit from different methods. We hope that eligible RCTs are warranted to verify the efficacy of the neuroendoscopic approach for HICH in the future.

## CONFLICT OF INTEREST

All authors certify that they have no affiliations with or involvement in any organization or entity with any financial interest (such as honoraria; educational grants; participation in speakers' bureaus; membership, employment, consultancies, stock ownership, or other equity interest; and expert testimony or patent‐licensing arrangements), or nonfinancial interest (such as personal or professional relationships, affiliations, knowledge, or beliefs) in the subject matter or materials discussed in this manuscript.

## INFORMED CONSENT

Informed consent was not required because no human participants involved in this study.

## Data Availability

The datasets generated and analyzed during the current study are available from the corresponding author on reasonable request.
